# Extracellular vesicle miRNAs from three-dimensional ovarian cancer in vitro models and their implication in overall cancer survival

**DOI:** 10.1016/j.heliyon.2025.e42188

**Published:** 2025-01-23

**Authors:** Nihar Godbole, Andrew Lai, Flavio Carrion, Katherin Scholz-Romero, Akhilandeshwari Ravichandran, Priyakshi Kalita-de Croft, Amy E. McCart Reed, Vaibhavi Joshi, Sunil R. Lakhani, Mostafa Kamal Masud, Yusuke Yamauchi, Lewis Perrin, John Hooper, Laura Bray, Dominic Guanzon, Carlos Salomon

**Affiliations:** a Translational Extracellular Vesicles in Obstetrics and Gynae-Oncology Group, Centre for Clinical Diagnostics, UQ Centre for Clinical Research (UQCCR), Royal Brisbane and Women's Hospital, Faculty of Medicine, The University of Queensland, Herston, QLD 4029, Australia; b UQ Centre for Extracellular Nanomedicine, Faculty of Medicine, The University of Queensland, Herston, QLD 4029, Australia; c Departamento de Investigación, Postgrado y Educación Continua (DIPEC), Facultad de Ciencias de la Salud, Universidad del Alba, Santiago 8320000, Chile; d Centre for Biomedical Technologies, Queensland University of Technology, 60 Musk Ave., Kelvin Grove, QLD 4059, Australia; e School of Mechanical, Medical and Process Engineering, Faculty of Engineering, Queensland University of Technology, Brisbane City, QLD 4000, Australia; f ARC Training Centre for Cell and Tissue Engineering Technologies, Queensland University of Technology, Kelvin Grove, QLD 5059, Australia; g UQ Centre for Clinical Research, Faculty of Medicine, The University of Queensland, Herston, QLD 4029, Australia; h Pathology Queensland, The Royal Brisbane and Women's Hospital, Herston, QLD 4029, Australia; i Australian Institute for Bioengineering and Nanotechnology (AIBN), The University of Queensland, St Lucia, QLD 4072, Australia; j Mater Research Institute, Translational Research Institute, Woolloongabba, QLD 4102, Australia

**Keywords:** Ovarian cancer, Cell culture models, Extracellular vesicles, Three-dimensional culture

## Abstract

Ovarian cancer is the most common gynaecological malignancy and the seventh most diagnosed cancer in females worldwide. Currently, it is the sixth leading cause of cancer related mortality among patients globally. The heterogenous origin of the disease and unambiguous nature of the clinical symptoms leading to delayed detection has been one of the key reasons for increasing mortality. Hence new approaches are required to understand the biology of ovarian cancer, where the use of cell culture models that mimic the physiology of the disease is fundamental. Cell culture serves as a crucial in vitro tool, contributing to our comprehension of various aspects of cell biology, tissue morphology, disease mechanisms, drug responses, protein production, and tissue engineering. A significant portion of in vitro studies rely on two-dimensional (2D) cell cultures, however, these cultures present notable limitations, for example disruptions in cellular and extracellular interactions, alterations in cell morphology, polarity, and division mechanisms. Recently, extracellular vesicles have been identified as crucial players in cell biology as part of the communication system that cancer cells use to metastasize. We optimized and compared three-dimensional (3D) culture of ovarian cancer cells lines (SKOV-3 and OVCAR-3) with two-dimensional models based on their protein and miRNA content. We further investigated whether extracellular vesicles from these models reflect changes in cancer cells, and aid in the identification of overall survival in women with ovarian cancer.

## Graphical abstract

A schematic representation of the study design, which involved the development and characterization of the ovarian cancer 3D cell culture models using SKOV-3 and OVCAR-3 cells and isolation of the extracellular vesicles from these models. These EVs were further used to compare the changes in the EV associated miRNA when compared to EV isolated from 2D monolayer cultures. Lastly, the EVs isolated from the 3D models were used for performing the comparative analysis to identify association of EV-associated miRNAs with the overall patient survival.

**Figure:**
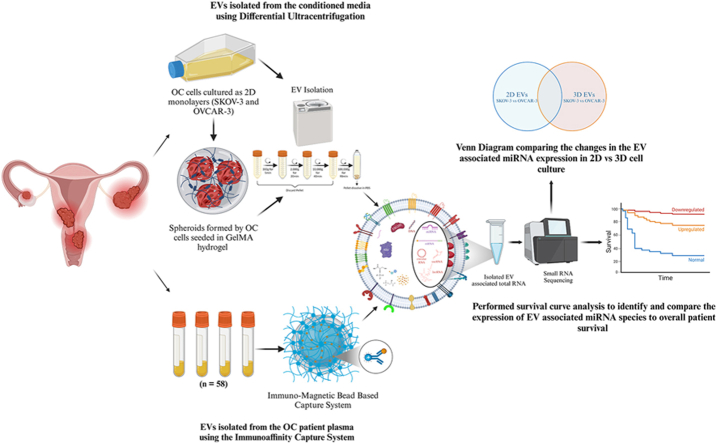

## Significance

Our study focuses on optimizing three-dimensional cell models to better mimic ovarian cancer physiology, exploring the role of extracellular vesicles in metastasis, and potentially identifying factors affecting overall survival in ovarian cancer patients. This research has clinical significance in advancing our understanding of ovarian cancer biology and potentially improving diagnostic and therapeutic strategies.

## 1 Introduction

Ovarian Cancer (OvCa) is the most common gynaecological malignancy and globally is the seventh most commonly diagnosed cancer in females [1]. Currently, it is the sixth leading cause of cancer-related mortality in patients worldwide [1]. According to the 2022 Cancer Fact Sheet released by Global burden of Cancer (GLOBOCAN) 324,398 new cases of OvCa and 206,839 deaths were registered worldwide [1,2]. OvCa is a heterogenous disease and histologically categorized into epithelial cancer which accounts for the majority of the cases (about 90 %), while the remaining 10 % of OvCa cases comprise of other non-epithelial cancers including germ cell tumors and sex chord stromal tumors [3]. Delayed diagnosis, resistance to chemotherapy, high metastasis, and heterogeneity are some of the major causes of OvCa related mortality worldwide [4]. The heterogeneous nature of the disease has limited many researchers from developing a uniform strategy for the early detection and screening of OvCa [5,6]. Moreover, despite the relentless efforts to develop innovative chemotherapeutic agents and precision therapies, the daunting challenges of resistance and recurrence in OvCa continue to hinder clinical efforts [7,8]. Therefore, gaining insight into cell-to-cell interactions within the tumour microenvironment in OvCa that can identify new therapeutic targets in a physiological context remains of utmost importance.

Extracellular vesicle (EV) refers to a heterogenous population of membrane-derived vesicles released by cells into the extracellular spaces and participates in cell-to-cell communication with neighbouring and distant cells [9]. Initially, these heterogeneous vesicles were believed to be the waste material of cells. However, continued research and substantial evidence indicated that these EVs are critical in cell-cell communication and responsible for regulating numerous functions on their target cells. The membrane of EVs consists of a phospholipid bilayer, and based on their biogenesis and size, they can be further categorized into small extracellular vesicles (sEVs) (≤200 nm) and large EVs (>200 nm) [10,11]. Exosomes are defined as a type of small EVs (around 30–150 nm) released from the endosomal compartment of the cells; other EVs such as microvesicles and ectosomes are released by the outward budding of the plasma membrane and are 30–1000 nm in size [10,[12], [13], [14]].

EVs encapsulate bioactive cargo or tissue-specific signalling molecules, such as nucleic acids and proteins. Upon their release, they can regulate the functions of recipient cells [14]. The function of EVs to transport biologically active cargo from inside cells to recipient cells also suggests their role in carcinogenesis by forming a premetastatic niche [15]. Therefore, several recent studies have used this property of EVs as potential biomarkers in cancer. EVs are also predicted to be involved in intercellular communication between tumor and stromal cells and are known to play a relevant role in numerous pathophysiological activities such as angiogenesis, tumour progression, cell survival, inflammation and coagulation [16,17].

Various physiologically relevant models are currently used in the laboratory setting to study the role of EVs in OvCa, including two-dimensional (2D) monolayers, animal models and 3D models [18]. Therefore, the biogenesis, packaging, and release of EVs are highly dependent and sensitive to the cellular microenvironment and depend on the in-vitro and in-vivo modelling systems used [18,19]. A significant number of studies isolating EVs from cell conditioned media (CCM) use conventional 2D cultured cells as a model system [18,20]. However, these cells exhibit a flat morphology, display apical-basal polarity, and tend to have altered gene expression and miRNA splicing [[21], [22], [23], [24], [25]]. Therefore, the limitations of 2D cultures in representing the complex physiological tumor microenvironment (TME) may overshadow their advantages. Consequently, it is highly plausible that the EV contents isolated from CCM of 2D cultures differ from those of 3D culture models. A pivotal solution would be the development of a 3D organotypic model that replicates tissue complexity and addresses the challenges associated with in-vivo experiments [26]. The concept of 3D cell cultures was first suggested by Bissell et al., in 1980, highlighting their potential to better mimic tissue-specific microenvironments [27]. In an ideal 3D cell culture model, cultured cells should proliferate, differentiate, and more accurately simulate pathophysiological microenvironments. Therefore, in this study, our objective is to optimize and establish a standardized technique for developing 3D culture models for ovarian cancer cells using Gelatin Methacryloyl (GelMA)-based hydrogels. We focused on the characterization of EVs from these optimized 3D models and, through Next Generation Sequencing (NGS), identified a set of common and unique miRNAs that may provide better insights into the processes occurring in patients with ovarian cancer.

## 2 Materials and methods

### 2.1 Ethics statement and quality management system

This study was performed in accordance with the Declaration of Helsinki. The overall analysis was approved by the Ethics committee of the University of Queensland and Mater Hospital (2023/HE002226), Human Research Ethics Committees at The University of Queensland (2005000785), and Royal Brisbane and Women's Hospital (RBWH 2005/022). All patients provided written informed consent. All experimental procedures were conducted within an ISO17025 accredited (National Association of Testing Authorities, Australia) research facility. All data were recorded within a 21 Code of Federal Regulation (CFR) part 11 compliant electronic laboratory notebook (Lab Archives, Carlsbad, CA 92008, USA).

### 2.2 Cell culture

Human OvCa cell lines of epithelial origin – SKOV-3 and OVCAR-3 were used. The cell lines were validated for the absence of mycoplasma contamination and the cell line authentication is provided in the supplemental material (Supplementary Fig. 1 and Fig. 2). Both the cell lines were maintained in culture media - RPMI 1640 media ([+] L-Glutamine & [−] Phenol Red, Gibco™, Life Technologies Corporation, Grand Island NY, USA Cat. No. 11835030) supplemented with 10 % heat-inactivated Foetal Bovine Serum (FBS) (PAA Laboratories Pty Ltd., Morningside, QLD, Australia) and 1000 U/mL antibiotic-antimycotic, (Gibco, Life Technologies, Carlsbad, CA, USA). The cells were cultured as adherent monolayers in T175 flasks and maintained in a CO_2_ incubator supplied with air containing 5 % CO_2_.

### 2.3 Cell encapsulation

On the day of encapsulation, the cells cultured in T175 were washed twice using phosphate-buffered saline (PBS) (Cat No. 14190144) and were later trypsinized using TrypLE™ Express ([−] Phenol Red) (Life Technologies Corporation, Grand Island NY, USA Cat. No. 12604013). The cells were then counted using an automated cell counter – Countess 3 (Catalog No. AMQAX2000 Thermo Fisher Scientific) and a desired number of cells were then transferred to a new 1.5 ml autoclaved Eppendorf tubes. The cells were then centrifuged at 300g for 5 min, the supernatant was discarded, and the pelleted cells were then encapsulated using GelMA biomaterials.

### 2.4 Encapsulation using GelMA

The precursor solution was made by mixing 5 % Porcine skin GelMA (Gelomics Pty Ltd., Kelvin Grove, QLD, AUS) with 0.15 % of photoinitiator lithium phenyl-2,4,6-trimethylbenzoylphosphinate (LAP), then sterile PBS was added to make up the final volume. The solution was then added to 50,000 pelleted cells and mixed thoroughly. This cell suspension was then cast as 20 μl droplets on a Teflon surface which was then subjected to gelation for 1 min using photo cross-linking at 405 nm light exposure using a Luna Crosslinker™ (Gelomics Pty Ltd., Kelvin Grove, QLD 4059, Australia). These gels were then transferred to the 48-well plate (Corning Life Science, Tewksbury, MA, USA) containing 1 ml of culture media, and the plate was maintained in a CO_2_ incubator supplied with air containing 5 % CO_2_ (Supplementary Fig. 3).

### 2.5 Phase contrast microscopy

The phase contrast images of the SKOV-3 and OVCAR-3 cell-laden gels were captured using a Nikon Eclipse Ts2 Routine Inverted Microscope. The images were captured to observe the growth and proliferation of cells inside the hydrogels on days 1, 3, 7 and 9 post-encapsulation.

### 2.6 Cell viability assay

The staining solution was made by mixing a stock solution of Fluorescein diacetate (FDA) (5 mg/ml) and propidium iodide (PI) (2 mg/ml) in 1 ml PBS. The gels were washed using sterile PBS for approximately 30–45 s before staining them with FDA/PI. The images were then captured using the stereoscope Nikon Fluorescence SMZ25 to observe the live and dead cells inside the gels. These images were taken at 1.5X magnification as multi-step z-stacks. The image sequences were then processed using ImageJ software.

### 2.7 DNA quantification using Quant-iT™ PicoGreen™

The cell-laden hydrogels were lysed using proteinase K by incubating overnight at 37°C. The standards were prepared using the serial dilutions of Lambda (λ) DNA standard and the total DNA quantification for the sample and standards was performed using the manufacturer's procedure provided with Quant-iT™ PicoGreen™ dsDNA Assay Kit (Catalog No. P7589 Thermo Fisher Scientific). The DNA from standards and samples was quantified by fluorescence measured at excitation: 480 nm and emission: 520 nm using BMG-POLARstar fluorescent plate reader. A final DNA concentration for samples was then calculated using a standard curve.

### 2.8 Cryoembedding and cryosectioning

The hydrogels were fixed using 4 % paraformaldehyde (PFA) and stored in a fresh plate before cryo-embedding. For cryo-embedding, the fixed gels were transferred to a plate containing 30 % sucrose solution and once settled at the bottom they were transferred to a new plate containing a 1:1 mixture of 30 % sucrose and OCT – a cryo-embedding matrix (Polarstat: PEL27301-3-4OZ) and incubated at 4°C for overnight. The gels were then transferred to cryo-embedding moulds and were topped up with the OCT compound and snap frozen using liquid nitrogen and stored at −20°C. The cryosectioning for these samples was performed using CryoStar™ NX50 Cryostat (Thermo Fisher Scientific). Samples were sectioned 10 μm thick and collected on Polysine slides (Thermo Scientific, item no. J2800AMNZ) and were stored at −20°C.

### 2.9 Haematoxylin and eosin (H&E) staining

The slides containing the cryosections were thawed at room temperature and were rinsed in PBS for 5 min to wash off the OCT. The sections were delineated using a DAKO pen (Agilent, cat. No. S200230-2). The residual gel covering the spheroids was digested using 0.1U collagenase. The sections were examined under a microscope. The slides were passed on to the Central Analytical Research Facility (CARF) at the Queensland University of Technology (QUT) for the H&E staining. The stained slides were scanned using Leica SCN400 High Throughput Slide Scanner.

### 2.10 Immunofluorescence

Slides containing the cryosections were thawed at room temperature and were rinsed in PBS for 5 min to wash off the OCT. The sections were delineated using a DAKO pen (Agilent, cat. No. S200230-2). The residual gel covering the spheroids was digested using 0.1U collagenase. The slides were washed twice using wash buffer (containing 0.05 % Tween-20) and the spheroids were permeabilized using 0.1 % Triton-X-100. The spheroids were blocked for 30 min using blocking buffer containing 1 % goat serum and were incubated overnight with primary antibodies (Abcam - Anti-E Cadherin – ab231303 (1:250); Anti-N Cadherin – ab98952 (1:200); Anti-Vimentin – ab8978 (1:100); and Anti-Pan Cytokeratin – ab86734 (1:250)) at 4°C. Following incubation, the slides were washed three times using wash buffer and were incubated with Alexa Flour 488 goat-anti mouse – A11001 (1:200) secondary antibody for 1 h in the dark. The slides were washed three times using wash buffer and mounted using coverslips. The slides were observed under the fluorescence microscope.

### 2.11 Cell recovery from GelMA hydrogels

The cell recovery from GelMA hydrogels was performed using the LunaGel™ - Cell Recovery Kit (Gelomics Pty Ltd.) according to the manufacturer's protocol.

### 2.12 Extracellular vesicle isolation and characterisation

The EVs were isolated from the serum-free media. Therefore, on day 7 the gels from 48 well plates containing FBS supplemented culture media were first washed using sterile PBS and then transferred to a fresh plate with the serum-free culture media. The spheroids were incubated for 48 h before the conditioned media was collected. The CCM was collected on day 9 and was centrifuged at 300g for 10 min. This was followed by differential centrifugation at 2,000g for 20 min and 10,000g for 40 min to remove all the cell debris. Finally, the supernatant was ultracentrifuged at 100,000g for 90 min with the Type 70.1 Ti fixed angle rotor (Beckman Coulter, CA, USA) to pellet the EVs. The obtained pellet was dissolved in filtered PBS and stored at −80°C. The EVs were then characterised according to the minimal information for studies of extracellular vesicles (MISEV) guidelines; based on their size distribution (Nanoparticle Tracking Analysis), morphology (Transmission Electron Microscopy), and abundance of the protein associated with these EVs – CD63, CD81, and CD9 (ExoView R100, NanoView Biosciences) [28]. For the isolation of EVs from the plasma samples a bead-based immunoaffinity capture (EXO-NET) method that utilizes antibody coated microbeads specific to surface markers present on EVs was used. EVs were captured using EXO-NET Pan-Exosome Capture beads (INOVIQ Ltd, Australia) following the manufacturer's protocol. The beads and the samples were equilibrated to room temperature. An aliquot of the plasma sample was mixed with the beads in 1:10 ratio and incubated for 15 min at room temperature. The mixture was then placed on the magnetic rack and was incubated until the supernatant became clear. The supernatant was carefully removed, and the pellet was washed using PBS. Notably, no serial centrifuge, such as at 300g, 2600g, 16000g, was performed to remove cell debris, apoptotic bodies, and microvesicles before applying the EXO-NET microbead.

### 2.13 NanoParticle tracking analysis

Nanoparticle tracking analysis (NTA) was performed using a Nanosight NS500 linked to an autosampler. Prior to sample analysis, the instrument's performance was validated using a 100 nm latex size standard (Malvern Scientific). All samples were diluted in DPBS to achieve an ideal particle/frame count of 20–100 in a final volume of 1 mL. For each measurement, 3 videos of 60 s were recorded using the camera level setting 13. The captured videos were then analysed by the NTA 3.4.4 software with the detection threshold set at 5. The data was exported as an excel file and the different populations of EVs were classified according to size as i) EVs (≤200 nm) and (ii) Large EVs (≥200 nm).

### 2.14 Transmission electron microscopy

EVs isolated by differential ultracentrifugation were assessed by transmission electron microscopy. EV pellets were fixed in 3 % (w/v) glutaraldehyde and 2 % paraformaldehyde in cacodylate buffer, pH 7.3. Five microlitres of sample was then applied to a continuous carbon grid and negatively stained with 2 % uranyl acetate. The samples were examined in an FEI Tecnai 12 transmission electron microscope (FEI™, Hillsboro, Oregon, USA).

### 2.15 Single particle analysis

Particle distribution and concentration were obtained using ExoView R100 (Nanoview Biosciences) analysis as previously described [29]. Briefly, ExoView R100 uses an antibody (viz. anti-CD81, anti-CD63, and anti-CD9) coated microarray chip (EV-TETRA-P) that binds to the EV associated surface markers, ensuring their expression on the measured particles. Using single-particle interferometric reflectance imaging, ExoView is highly sensitive and can accurately measure a single EV particle (∼50 nm diameter). A total of 2.5 μg of EVs were diluted in 500 μl incubation solution and a 40 μl aliquot was dispensed on the chip for overnight incubation. Following incubation, the chip was washed three times using the incubation solution and further incubated for 1 h with CD81, CD63, and CD9 antibodies in 1:1200 dilution. Afterwards, the chip was washed using the incubation solution followed by wash solution and then analysed (ExoView R100). The chips were prepared in triplicates and the whole chip was scanned for particle analysis.

### 2.16 RNA isolation

The total RNA isolation from the cells and EVs was performed using miRNeasy Mini Kit (QIAGEN, Cat. No. 217004) and TRIzol™ reagent (Thermo Fisher Scientific, cat. No. 15596018) according to manufacturer's protocol. The total RNA for the EVs isolated from the patient samples was extracted using Norgen Exosomal RNA Isolation kit (NORGEN, Cat. No. SKU 58000) The RNA concentration was then measured on a Qubit™ (Thermo Fisher Scientific), using the Qubit™ RNA High Sensitivity and Qubit™ microRNA Assay Kits.

### 2.17 Small RNA sequencing

Sequencing libraries were generated using the NEXTflex™ Small RNA-Seq Kit v3 with UDIs according to the manufacturer's instructions. The small RNAs within the total RNA were quantified using the Qubit™ microRNA Assay Kits, and approximately 5–10 ng of small RNA was used as input for library preparation. The samples underwent 3 prime ligation, 5 prime ligation and reverse transcribed, where 1/4th adapter was used during the ligation steps. Afterwards, the RNA samples underwent PCR (25 cycles) containing primers with unique barcode sequences to allow pooling of samples into groups of 96, followed by library size selection using gel electrophoresis. Finally, the DNA libraries were eluted from the gel pieces overnight with shaking, followed by a bead cleanup. The final library was quantified using the KAPA Library Quantification Kit (Roche) and library size was determined using the Tapestation High Sensitivity D1000. A total of 96 samples were pooled in equimolar quantities, and the final pooled library was sequenced using the NextSeqs 500 and 75 cycles High Output kit (single end for 75 cycles).

### 2.18 Patient derived specimens

Case control study design was undertaken using plasma samples (n = 58) from women with OvCa patients from the Mater Hospital biobank (Brisbane, Australia) (Table 2). Samples were collected between 2018 and 2024 using the same standardized operating procedure for blood collection. Women were consented for blood draw (10 mL in EDTA) before surgery for suspected ovarian adenocarcinoma. Plasma was made by centrifugation at 2500×*g* for 10 min and 0.5 mL aliquots were banked at −80°C. Study subject coded data included patient age, pathological diagnosis (e.g. benign cystadenoma, borderline tumour, primary ovarian carcinoma including differentiation and grade, or metastatic carcinoma including classification) that was confirmed by a board-certified pathologist with expertise in gynaecologic pathology, clinical stage, whether the patient was optimally debulked (yes or no), interval from blood collection to follow up assessment, and clinical outcome at follow up.

### 2.19 Statistical analysis

The small RNA sequencing data was processed using Cutadapt (version 4.3) [30] to remove adapters, then analysed to identify miRNAs using the miRDeep2 software (version 2.0.1.2) [31]. Subsequently, the identified miRNAs were normalised and statistically analysed (Wald test with Benjamini and Hochberg correction) using the DESeq2 package in R (version 1.34.0) [32], to find differences between 2D and 3D models. In addition, the normalised miRNA counts were statistically analysed using Kruskal-Wallis test to find differences between normal, benign and ovarian cancer stages. Afterwards, statistically significant differences were subjected to multiple comparison analysis using the Wilcoxon test.

The stratification of patients into low and high expression groups was performed using maximally selected rank statistics. Afterwards, survival outcomes were compared using Kaplan–Meier analysis with log-rank test, using the survminer (version 0.4.9) and survival (version 3.2–13) R packages [33]. Gene ontology over-representation analysis was performed using the rbioapi R package (version 0.8.0) [34], accessing the miEAA web service (version 2.1) [35]. The R statistical software version 4.1.3 (R Foundation for Statistical Computing, Vienna, Austria) was used for analyses, while ggplot2 (version 3.4.4) [36], ggpubr (version 0.4.0), ggpattern (version 1.0.0–0) and gt (version 0.10.1) R packages was used for visualisation and table generation. P-values <0.05 were considered statistically significant unless otherwise noted.

## 3 Results

### 3.1 Characterization of 3D cell culture model

**Fig. 1:**
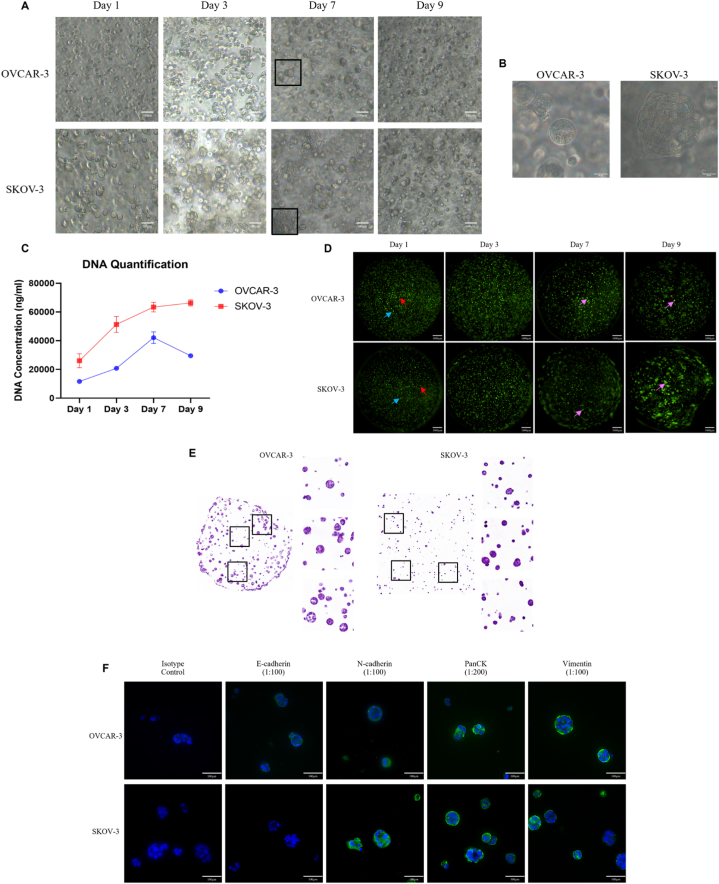
Comprehensive characterisation of 3D cell culture model for its biocompatibility towards the OC cells thereby observing the growth, development, and histology of the cells inside the hydrogel post-encapsulation. (A) Representative phase contrast images of cell laden hydrogels captured on day 1, 3, 7, and 9 post encapsulations at 4X objective using a phase contrast microscope. (B) Phase contrast images captured at 20X for the spheroids formed by OvCa cells on day 7 of post-encapsulation. (C) Quantifying the total DNA concentration of the GelMA encapsulated cells using Quant-iT PicoGreen assay as a representative for evaluating the cell growth. (D) Representative images captured using fluorescence microscope for assessing the viability of encapsulated OvCa cells using fluorescence-based double staining procedure of FDA/PI, the images were captured at 1.5X magnification as a multistep Z-stack. (E) Representative image of the cryosections of cell-laden hydrogels stained using haematoxylin and eosin stain. The slides were scanned using Leica SCN400 high throughput slide scanner and observed at 10X and 40X. (F) Representative images of staining patterns observed via immunofluorescence analysis performed on the cryosections of cell laden hydrogel for validating the distribution of EMT markers (E-Cadherin, N-Cadherin, PanCK and Vimentin). Data are presented as mean ± SD.

In this study, both cell lines were cultured as 2D monolayers under controlled conditions before encapsulating in GelMA hydrogels. Upon reaching 70 % confluency, adherent cells were encapsulated in the hydrogel. The phenotypic characterization of the spheroids involved monitoring the growth and proliferation of OvCa cells inside the hydrogels using phase contrast imaging, total DNA quantification, and fluorescence-based cell viability imaging. The spheroid formation of OvCa cells was observed using the phase contrast images captured at 4X and 20X. Notably, OVCAR-3 cells displayed a distinct spheroid formation on days 7 and 9 of post encapsulation while the SKOV-3-laden hydrogel exhibited a mixed population of spheroids and aggregated cellular structures hereafter referred to as multicellular aggregates (MCAs) (Fig. 1A). Moreover, the images captured using the 20X objective further elucidated the distinct morphology of the spheroids and MCAs of OVCAR-3 and SKOV-3 respectively (Fig. 1B). The proliferation of OvCa cells encapsulated in the hydrogel was evaluated by quantifying the total DNA using Quant-iT PicoGreen assay using the lambda DNA as a reference. Using Tukey's multiple comparison tests to analyse the changes over time in total DNA concentration, we observed a significant increase in the DNA content of both cell lines up to day 7 post-encapsulation. However, in contrast to SKOV-3, the total DNA content in OVCAR-3 cells significantly declined from day 7 to day 9 (Fig. 1C). To assess the viability of the encapsulated cells, we used an FDA/PI fluorescence-based double staining procedure, and the images were captured using a fluorescence microscope (Fig. 1D). Based on these images, we observed a significantly higher number of viable cells fluorescing green (indicated by the blue arrow) compared to red fluorescing dead cells (indicated by the red arrow) for both cell lines. Consistent with observations made using phase contrast microscopy, viable spheroids, and MCAs (indicated by the pink arrow) were visible in SKOV-3-laden hydrogels on days 7 and 9.

### 3.2 Histological characterization of spheroids

Based on the phenotypic characterization of OvCa spheroids, we observed proliferation and viability of the cells inside the hydrogel suggesting the biocompatible nature of the hydrogel. Furthermore, the ability of these OvCa cells to form spheroids and multicellular aggregates (MCAs) emphasizes the nature of cancer cells to proliferate, aggregate, and differentiate. Next, these spheroids were histologically characterized using haematoxylin and eosin (H&E) staining and immunofluorescence (IF) against epithelial-mesenchymal transition (EMT) markers. Based on the H&E staining, we observed multinucleated centres (stained purple) present inside a spherical cytoplasm (stained pink), confirming the spheroidal morphology of both the cell lines inside the hydrogel (Fig. 1E). Immunofluorescence analysis was performed on cryosections of the hydrogel to validate the expression of OvCa-associated EMT markers, including E-cadherin, N-cadherin, pan-cytokeratin (PanCK), and vimentin (Fig. 1F). This analysis aided in further histological characterization of the OvCa spheroids, wherein the OVCAR-3 spheroids exhibited minimal expression of the epithelial surface marker E-cadherin, while SKOV-3 spheroids were completely devoid of its expression. In contrast, we observed strong staining for the mesenchymal marker N-cadherin in SKOV-3 spheroids compared to minimal staining in OVCAR-3 spheroids, while both cell lines also exhibited strong expression of the epithelial marker PanCK. Furthermore the expression of vimentin, a mesenchymal marker evident in the spheroids of both the cell lines, had stronger staining in OVCAR-3 compared to SKOV-3, the later having a mixed population of spheroids with strong to intermediate staining.

### 3.3 Characterization of enriched extracellular vesicles

**Fig. 2:**
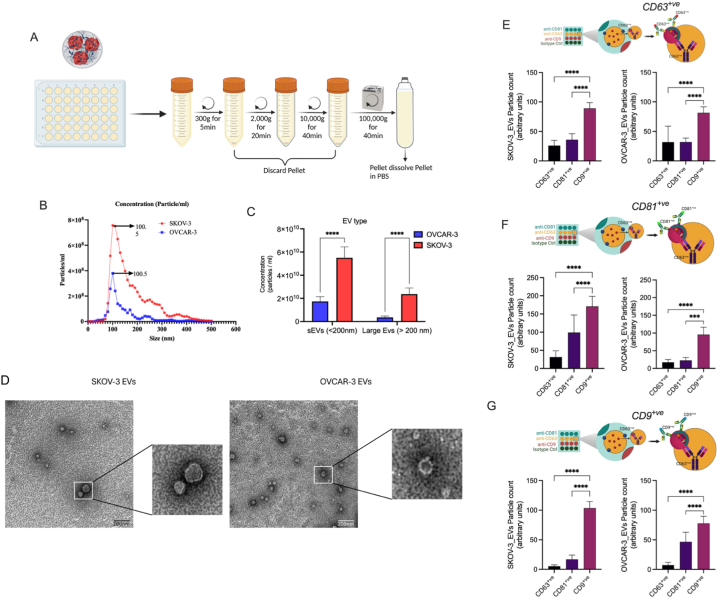
Characterising the enriched extracellular vesicles isolated from a 3D cell culture model of OvCa cell lines based on their size, concentration (using nanoparticle tracking analysis), morphology (using transmission electron microscopy), and surface markers (using ExoView R100) according to guidelines provided by MISEV 2018. (A) A schematic representation of the methods used for isolating the EVs from the cell conditioned media harvested from OvCa 3D cells. (B) Concentration of total EVs isolated from the SKOV-3/3D and OVCAR-3/3D cells analysed using NTA, the average EVs size was identified to be ∼100 nm. (C) represents the heterogenous population of EVs – small EVs (≤200 nm) and large EVs (>200 nm) isolated from OvCa 3D cell culture. The statistical significance was evaluated using Sidak's multiple comparison test of two-way ANOVA, (D) Representative images captured at 20X using a transmission electron microscope for validating the morphology of the EVs isolated from OvCa 3D cell culture. (E–G) The fluorescent particle count for the OvCa/3D derived EVs captured on the tetraspanin (CD63, CD81, and CD9) spot and validated using fluorescent antibodies against CD63/CD81/CD9 analysed using ExoView R100 to characterise the EVs specific surface markers. ∗∗∗ (P ≤ 0.001), ∗∗∗∗ (P ≤ 0.0001).

The hydrogels were placed in the serum free media for 48 h for the isolation of the EVs. The EVs were isolated through a combination of differential centrifugation and ultracentrifugation and further characterized based on size, morphology, and surface markers, following the recommendations of the International Society of Extracellular Vesicles (ISEV, [28]) (Fig. 2A). The size distribution of the heterogeneous population of EVs was determined using nanoparticle tracking analysis, with a peak around 100 nm for both SKOV-3 and OVCAR-3-derived EVs (Fig. 2B), suggesting an enrichment of small EVs. Furthermore, we subcategorized the concentration of total EVs from the 3D models based on their size as small (≤200 nm) and large (>200 nm) EVs (Fig. 2C). The concentration of EVs from SKOV-3 spheroids was significantly higher compared to OVCAR-3 spheroids, with significantly higher enrichment of small EVs compared to large EVs (Fig. 2C). Morphological analysis performed using TEM revealed cup-shaped vesicles enclosed by a lipid membrane (Fig. 2D). To investigate the heterogeneity of EVs secreted from 3D ovarian cancer models, we performed a single-vesicle analysis using ExoView R100 (Nanoview Biosciences) and the EVs were captured using tetraspanin-specific antibodies. The ExoView analysis indicated the presence of CD63 (Fig. 2E), CD81 (Fig. 2F), and CD9 (Fig. 2G) on the captured vesicles. Notably, the population of EVs that were CD9^+ve^/CD63^+ve^ was significantly higher in both OVCAR-3 and SKOV-3 spheroids compared to CD63^+ve^/CD63^+ve^ and CD81^+ve^/CD63^+ve^ EVs (Fig. 2E). Similar results were observed in the population of CD9^+ve^/CD81^+ve^ (Fig. 2F) and CD9^+ve^/CD9^+ve^ (Fig. 2G) compared to other EV populations. This data suggests that these spheroids were able to secrete heterogeneous population of EVs with a varying abundance of tetraspanin proteins.

### 3.4 Differential expression profiling of miRNAs in 3D and 2D cultures of ovarian cancer cells and their extracellular vesicles

To understand the significance of alterations in post-transcriptional gene expression control, crucial for cellular processes, we performed a comprehensive analysis of miRNA expression profiles in 3D versus 2D OvCa cell culture models and their secreted extracellular vesicles using small RNA sequencing. Utilizing SKOV-3 and OVCAR-3 spheroids as a reference for the different stages of ovarian cancer oncogenic transformation, we observed significant variations in miRNA expression.

**Fig. 3:**
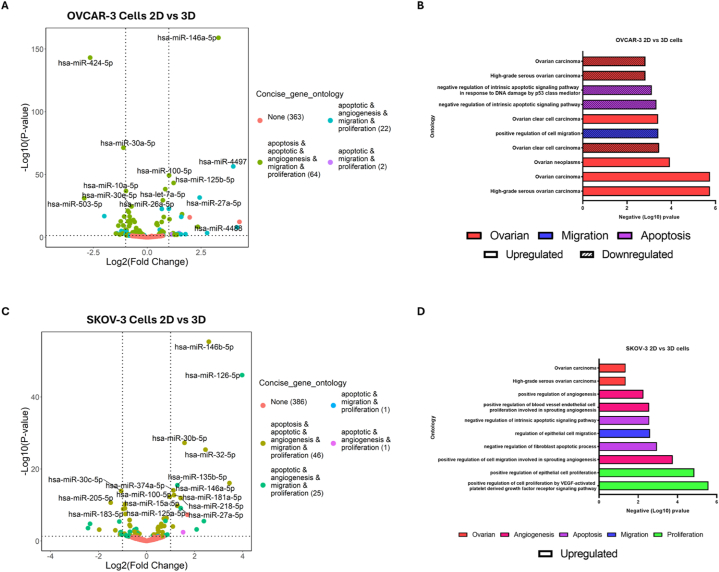
Comparison analysis of miRNA profiles in 2D versus 3D cell culture models of ovarian cancer. OvCa cells were cultured as either 2D monolayer or 3D culture models, and their miRNA profiles were analysed. The volcano plot graph showcases the expression of miRNAs between 3D and 2D cultures of OvCa cells, with the y-axis representing the log2 transformed fold change and the x-axis representing −log10 (p-value). The colors on the plot indicate the gene ontology analysis of genes targeted by the miRNAs. (A) Volcano plot between the miRNA profiles of 2D and 3D cultures of OVCAR-3 cells. (B) Ontology analysis of the upregulated and downregulated miRNAs in OVCAR-3 cells and their association with biological processes. (C) Volcano plot between the miRNA profiles of 2D and 3D cultures of SKOV-3 cells. (D) Ontology analysis of the upregulated and downregulated miRNAs in SKOV-3 cells and their association with biological processes.

In the OVCAR-3 spheroids, we identified 91 (Table 1) significant miRNAs that were differentially expressed, comprising of 43 upregulated and 48 downregulated miRNAs compared to 2D cultures (Fig. 3A). Whereas, in SKOV-3 spheroids, we identified significant differential expression of 76 miRNAs (Table 1) consisting of 37 upregulated and 39 downregulated miRNAs compared to 2D cultures (Fig. 3C). The gene ontologies (GO) for these dysregulated miRNAs from OVCAR-3 and SKOV-3 spheroids suggested their key role in the progression of OvCa by affecting the overall apoptosis, angiogenesis, proliferation, and migration (Fig. 3B–D).

**Table 1:** – miRNAs.

Downregulated in 3D			Upregulated in 3D		
miRNAs	Log2(FoldChange)	p-value	miRNAs	Log2(FoldChange)	p-value
OVCAR Cells					
hsa-miR-503-5p	−2.93	7.18 × 10^-32	hsa-miR-10396b-3p	4.28	4.89 × 10^-13
hsa-miR-424-5p	−2.65	6.95 × 10^-144	hsa-miR-4488	4.16	1.15 × 10^-8
hsa-miR-4454	−1.99	1.46 × 10^-17	hsa-miR-4497	3.99	3.20 × 10^-57
hsa-miR-3065-5p	−1.46	1.06 × 10^-3	hsa-miR-146a-5p	3.30	9.78 × 10^-160
hsa-miR-4517	−1.41	7.22 × 10^-4	hsa-miR-200a-5p	2.78	3.81 × 10^-4
hsa-miR-769-5p	−1.34	5.13 × 10^-10	hsa-miR-27a-5p	2.43	2.20 × 10^-32
hsa-miR-34b-5p	−1.27	6.07 × 10^-3	hsa-miR-1246	2.35	4.54 × 10^-9
hsa-miR-340-5p	−1.23	1.98 × 10^-13	hsa-miR-9985	1.97	1.33 × 10^-16
hsa-miR-450a-5p	−1.20	9.38 × 10^-6	hsa-miR-200b-5p	1.77	3.40 × 10^-3
hsa-miR-33a-5p	−1.14	9.26 × 10^-4	hsa-miR-1260b	1.61	3.98 × 10^-19
OVCAR EVs					
hsa-miR-339-5p	−5.53	2.99 × 10^-5	hsa-miR-423-5p	1.84	4.81 × 10^-14
hsa-miR-4454	−4.05	3.00 × 10^-4	hsa-miR-183-5p	1.38	3.09 × 10^-5
hsa-miR-151a-5p	−1.78	1.41 × 10^-5	hsa-miR-4466	1.14	2.23 × 10^-2
hsa-miR-99b-5p	−1.62	1.02 × 10^-3	hsa-let-7b-5p	1.11	9.48 × 10^-25
hsa-miR-10400-5p	−1.58	5.54 × 10^-3	hsa-miR-193b-5p	1.10	2.55 × 10^-2
hsa-miR-27a-5p	−1.45	1.23 × 10^-2	hsa-miR-146a-5p	0.99	3.77 × 10^-2
hsa-miR-92a-1-5p	−1.16	2.25 × 10^-2	hsa-miR-125b-5p	0.98	1.48 × 10^-3
hsa-miR-484	−1.07	3.36 × 10^-2	hsa-miR-1246	0.97	1.45 × 10^-2
hsa-miR-196a-5p	−1.06	2.90 × 10^-2	hsa-miR-320d	0.85	2.07 × 10^-2
hsa-miR-663a	−1.03	3.16 × 10^-2	hsa-miR-21-5p	0.65	2.53 × 10^-7
SKOV Cells					
hsa-miR-548ah-5p	−2.44	2.61 × 10^-4	hsa-miR-126-5p	3.97	8.20 × 10^-47
hsa-miR-4517	−2.36	1.65 × 10^-5	hsa-miR-146a-5p	3.45	8.40 × 10^-17
hsa-miR-7976	−1.97	7.09 × 10^-4	hsa-miR-146b-5p	2.59	4.48 × 10^-56
hsa-miR-205-5p	−1.50	2.15 × 10^-11	hsa-miR-32-5p	2.45	4.05 × 10^-26
hsa-miR-130b-5p	−1.30	1.06 × 10^-3	hsa-miR-135a-5p	2.38	2.85 × 10^-6
hsa-miR-516a-5p	−1.14	3.93 × 10^-6	hsa-miR-1248	2.08	6.01 × 10^-4
hsa-miR-519a-5p	−1.10	1.41 × 10^-2	hsa-miR-9985	1.70	5.07 × 10^-8
hsa-miR-486-5p	−1.09	4.17 × 10^-3	hsa-miR-30b-5p	1.57	5.45 × 10^-28
hsa-miR-519a-2-5p	−1.08	1.45 × 10^-2	hsa-miR-891a-5p	1.52	3.72 × 10^-3
hsa-miR-520b-5p	−1.08	1.45 × 10^-2	hsa-miR-27a-5p	1.42	7.30 × 10^-10
SKOV EVs					
hsa-miR-30c-5p	−2.17	2.43 × 10^-13	hsa-miR-1246	4.86	9.82 × 10^-27
hsa-miR-27a-5p	−1.15	8.15 × 10^-3	hsa-miR-423-5p	2.19	1.07 × 10^-18
hsa-miR-425-5p	−1.04	1.84 × 10^-2	hsa-miR-125b-5p	1.57	1.71 × 10^-5
hsa-miR-339-5p	−0.96	1.42 × 10^-2	hsa-let-7b-5p	1.13	5.83 × 10^-9
hsa-miR-21-5p	−0.88	7.35 × 10^-13	hsa-miR-125a-5p	1.09	1.59 × 10^-4
hsa-miR-30a-5p	−0.81	2.01 × 10^-5	hsa-miR-320d	1.04	1.49 × 10^-2
hsa-let-7f-5p	−0.74	3.55 × 10^-4	hsa-miR-4488	0.89	1.20 × 10^-2
hsa-let-7i-5p	−0.69	1.63 × 10^-2	hsa-miR-30d-5p	0.85	1.19 × 10^-3
hsa-miR-589-5p	−0.65	4.18 × 10^-2	hsa-miR-26a-5p	0.68	2.37 × 10^-2
NA	NA	NA	hsa-miR-10a-5p	0.66	1.93 × 10^-4

**Table 2:** Clinical table.

	Stage 1 (N = 10)	Stage 2 (N = 10)	Stage 3 (N = 28)	Stage 4 (N = 10)	ANOVA p-value
PatientAge					
Mean (SD)	44.67 (14.63)	40 (3)	61.19 (15.53)	57.56 (11.83)	9.63 × 10^-3
Median	44	40	64	58	–
Min - Max	19–66	37–43	22–86	31–73	–
n	9	3	27	9	–
CA125					
Mean (SD)	405.71 (692.87)	2011.88 (2817.99)	839.88 (1049.61)	5702.33 (10273.68)	4.58 × 10^-2
Median	173	1175	334.5	828	–
Min - Max	37–1960	8–8400	8–4325	18–31000	–
n	7	8	26	9	–
RedBldCellCount					
Mean (SD)	4.38 (0.52)	4.8 (0.26)	385.55 (1079.03)	3.95 (0.68)	4.51 × 10^-1
Median	4.37	4.86	4.41	3.94	–
Min - Max	3.6–5.02	4.45–5.01	2.64–3056	2.72–4.76	–
n	9	4	8	9	–
Haemoglobin					
Mean (SD)	129.33 (9.64)	137 (4.97)	121.88 (23.7)	116.56 (11.18)	1.12 × 10^-1
Median	130	137	129	115	–
Min - Max	117–145	131–143	86–150	95–130	–
n	9	4	8	9	–
Haemocrit					
Mean (SD)	0.39 (0.03)	0.42 (0.01)	0.37 (0.07)	0.35 (0.03)	8.40 × 10^-2
Median	0.39	0.42	0.39	0.35	–
Min - Max	0.34–0.42	0.41–0.43	0.26–0.46	0.28–0.4	–
n	9	4	8	9	–
MeanCellVolume					
Mean (SD)	89 (5.87)	87.45 (4.37)	92.46 (5.86)	83.71 (25.54)	6.85 × 10^-1
Median	86.4	87.3	90.45	93.4	–
Min - Max	80.9–96	83.1–92.1	87.2–106	19–104.1	–
n	9	4	8	9	–
PlateletCount					
Mean (SD)	281.78 (121.04)	363.75 (59.91)	289.38 (61.42)	273.44 (150.4)	5.97 × 10^-1
Median	255	350	277.5	239	–
Min - Max	154–564	307–448	224–402	84–578	–
n	9	4	8	9	–
WhiteBldCellCount					
Mean (SD)	8.41 (1.68)	8.38 (2.88)	6.84 (1.73)	8.02 (4.78)	7.35 × 10^-1
Median	7.9	8.8	7.1	6.5	–
Min - Max	5.8–11.8	5.1–10.8	4–9.5	2.5–16.3	–
n	9	4	8	9	–
AbsoluteNeutrophil					
Mean (SD)	12.21 (20.5)	5.49 (1.92)	5.03 (1.6)	6 (5.23)	5.71 × 10^-1
Median	5.08	5.72	5.26	3.93	–
Min - Max	4.08–66.74	3.29–7.21	2.52–7.15	1.06–15.4	–
n	9	4	8	9	–
Ovarian cancer grade					
High	1	4	22	9	–
Low	2	2	6	0	–
Nulliparous					
No	6	5	7	6	–
Yes	3	3	1	0	–
Menopause					
Pre	5	4	2	0	–
Post	3	5	5	6	–
Previous chemotherapy					
Yes	1	1	5	6	–
No	8	7	4	3	–

**Fig. 4:**
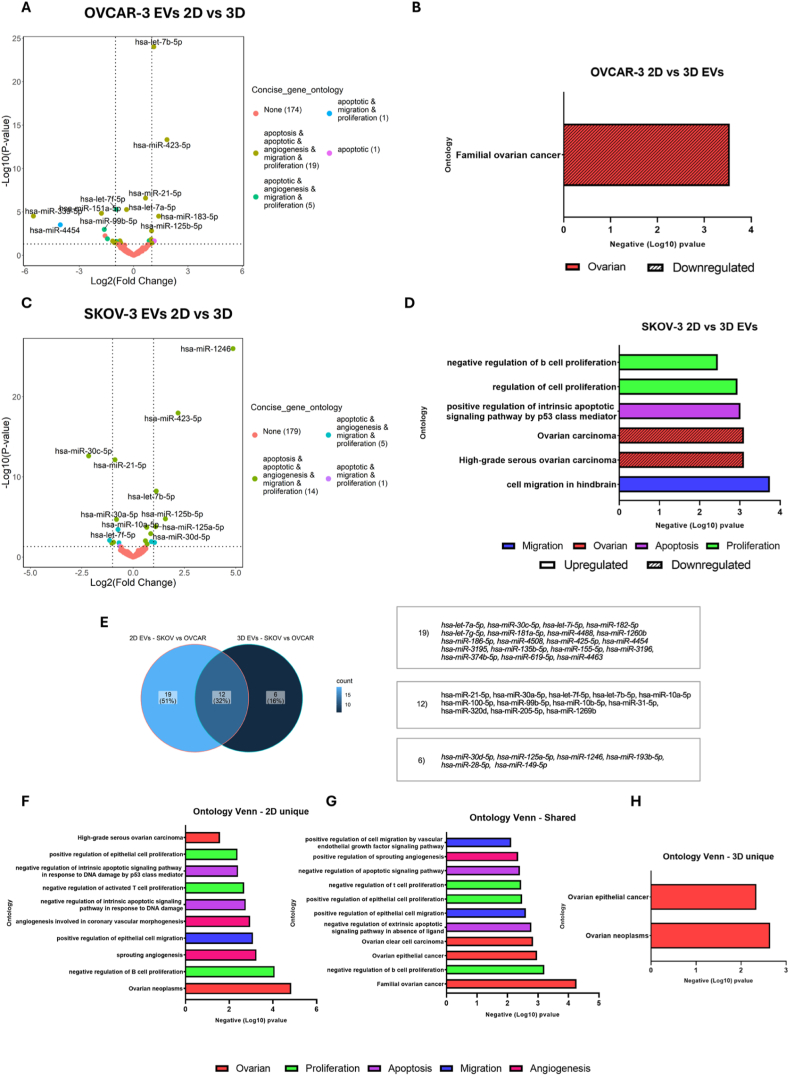
Analysis of differentially expressed miRNAs within extracellular vesicles (EVs) secreted from ovarian cancer cells cultured in 2D versus 3D environments. EVs were isolated from cells cultured in either 2D monolayer or 3D culture models, and their miRNA profiles were determined. The volcano plot graph illustrates the expression of miRNAs between 3D and 2D cultures of OvCa cells. On the graph, the y-axis represents the log2 transformed fold change, while the x-axis represents −log10 (p-value). The colors indicate the gene ontology analysis of genes targeted by the miRNAs. (A) Volcano plot between the miRNA profiles of 2D and 3D EVs from OVCAR-3 cells. (B) Ontology analysis of the upregulated and downregulated EV miRNAs in OVCAR-3 cells and their association with biological processes. (C) Volcano plot between the miRNA profiles of 2D and 3D EVs from SKOV-3 cells. (D) Ontology analysis of the upregulated and downregulated EV miRNAs in SKOV-3 cells and their association with biological processes. (E) Venn diagram showing the unique and common miRNAs within dysregulated EVs in 2D and 3D culture models. (F) Ontology analysis of the genes targeted by the unique miRNAs within EVs secreted from 2D OvCa cells. (G) Ontology analysis of the genes targeted by the common miRNAs within EVs secreted from both 2D and 3D OvCa cells. (H) Ontology analysis of the genes targeted by the unique miRNAs within EVs secreted from OvCa 3D cell culture models.

The EVs isolated from OVCAR-3 spheroids compared to 2D EVs had 27 differentially expressed significant miRNAs (Table 1), consisting of 10 upregulated and 17 downregulated miRNA species (Fig. 4A). While the EVs isolated from SKOV-3 spheroids had significant differential expression of 20 miRNAs (Table 1), with 11 upregulated and 9 downregulated miRNAs when compared to respective 2D EVs (Fig. 4C). The gene ontology for these dysregulated miRNAs suggested their role in angiogenesis, migration, and proliferation which are some of the notable traits for the progression of OvCa (Fig. 4B–D). Additionally, we analysed the changes in the expression profiles of the EV associated miRNAs to their cell culture counterparts. Based on the analysis we identified a total of 131 miRNAs significantly dysregulated (81 upregulated and 50 downregulated) in the EVs isolated from the 2D cell culture models. While for EVs isolated from 3D cell culture models, we identified 117 significantly dysregulated miRNAs (61 upregulated and 56 downregulated) (Supplementary Figs. 4A and B). For the 2D cell culture models, we detected 134 (63 %) species of unique miRNAs in the cells, while 12 (6 %) unique miRNAs were associated with the EVs. Moreover, we identified 67 (31 %) miRNAs that were shared between the EVs and the cells (Supplementary Fig. 4C). Similarly, for the 3D cell culture models we observed 146 miRNAs uniquely associated with the OvCa 3D cells, while only 6 unique miRNAs were detected in the EVs isolated from these 3D cells. Furthermore, we identified 47 (24 %) miRNAs that were shared between the EVs and the OvCa spheroids in 3D cell culture models (Supplementary Fig. 4D)

Subsequently, we assessed whether the observed differences in the miRNA expression profiles of the 3D versus 2D cell culture models were mirrored in their secreted EVs. The EVs derived from 3D spheroids exhibited distinct miRNA profiles compared to those from 2D cultures, with gene ontology analysis highlighting their roles in OvCa progression (Fig. 4F–H). Furthermore, several miRNAs were identified to exhibit consistent dysregulation patterns across different models (Fig. 4E), emphasizing their potential as diagnostic or prognostic markers for ovarian cancer.

Our findings underscore significant disparities in miRNA expression profiles between ovarian cancer cells cultured in 3D versus 2D and their secreted EVs, suggesting potential implications for disease progression. Further investigation into the expression of selected miRNAs within EVs isolated from OvCa patients is required to validate these findings.

### 3.5 Dynamic changes in EV miRNA profiles across ovarian cancer

To assess the physiological relevance of previously identified miRNAs in ovarian cancer (OvCa) cells cultured in both 3D and 2D models, we analysed a diverse cohort of patients, including those with ovarian cancer, benign conditions, and healthy individuals (Table 2). We observed significant differences in patient age distribution, with older patients being more prevalent in advanced cancer stages (III and IV). However, no significant differences were found in other clinical parameters, such as red blood cell count, hemoglobin levels, hematocrit, mean cell volume, platelet count, white blood cell count, and absolute neutrophil count.

We further examined OvCa grade, nulliparous status, menopause status, and previous chemotherapy history within each stage. This analysis provided key insights into disease status and patient demographics, contributing to a comprehensive understanding of disease progression across various stages of ovarian cancer.

EVs were isolated from plasma samples using a bead-based immunoaffinity capture technology, a reproducible method suitable for routine pathology laboratories. Antibodies binding to EV-associated proteins facilitated the capture. Targeted miRNA analysis was performed using small RNA sequencing, as outlined in the Methods section. The miRNA analysis was divided into three groups based on dysregulated expression, identifying miRNAs unique to or shared between EVs from 3D and 2D models (Fig. 4E). The categories include: Group A (unique to 2D-EVs), Group B (shared between 2D-EVs and 3D-EVs), and Group C (unique to 3D-EVs).

**Fig. 5:**
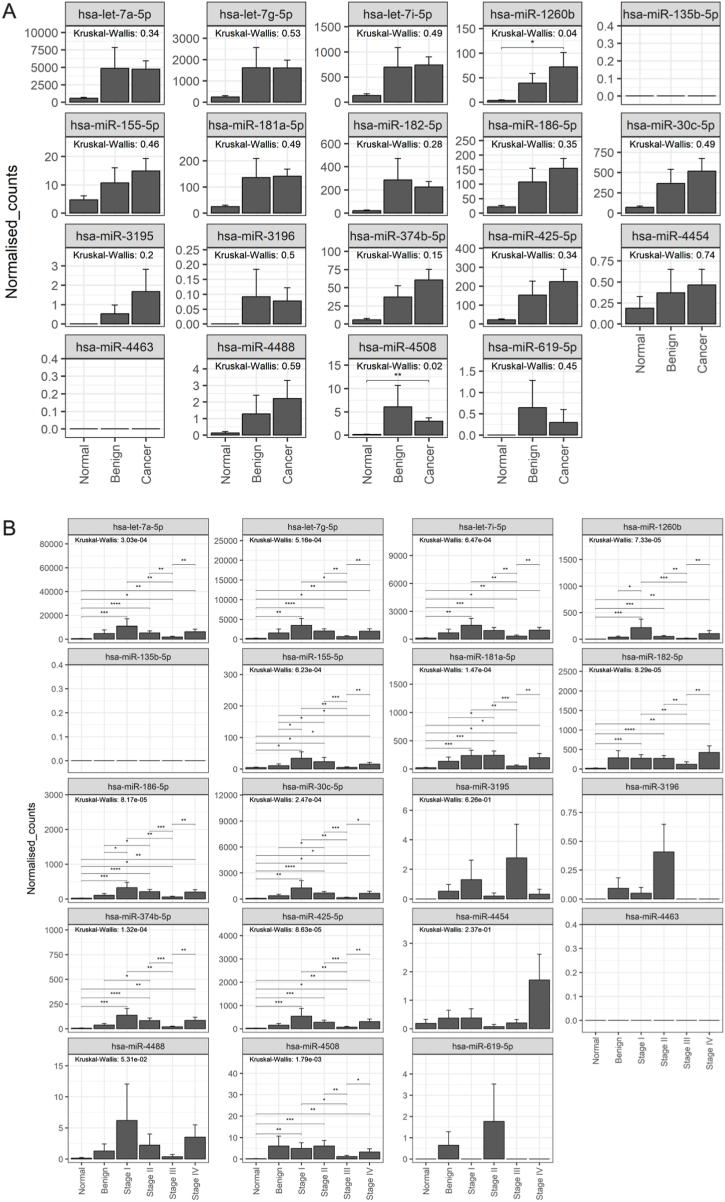
Analysis of miRNAs uniquely identified in extracellular vesicles (EVs) derived from ovarian cancer cells cultured in a 2D monolayer. EVs were isolated from plasma samples obtained from patients with OvCa, benign disease, and healthy controls. Targeted miRNA sequencing was conducted on selected groups of miRNAs. Panel (A) shows the miRNA expression across the patient groups, while panel (B) displays the miRNA expression in the groups subclassified by the stage of OvCa. Data are presented as mean ± SE. ∗ (P ≤ 0.05), ∗∗ (P ≤ 0.01), ∗∗∗ (P ≤ 0.001), ∗∗∗∗ (P ≤ 0.0001).

**Fig. 6:**
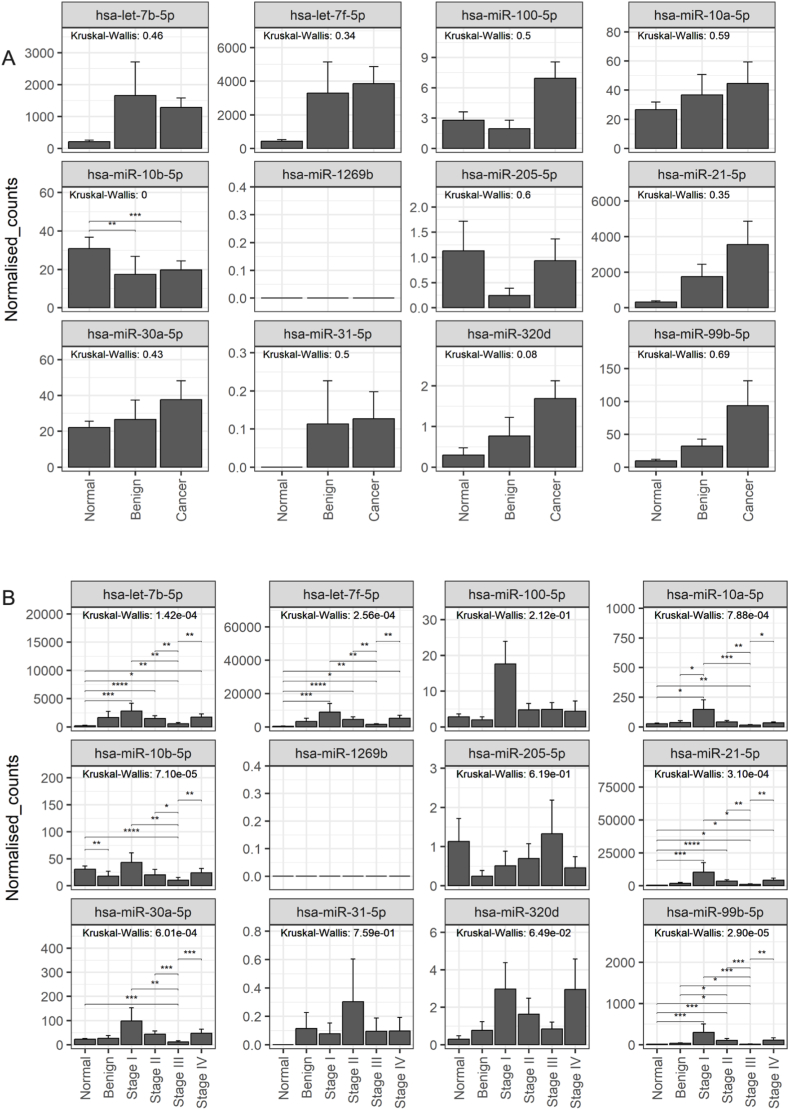
Analysis of miRNAs commonly identified in extracellular vesicles (EVs) derived from ovarian cancer cells cultured in a 2D monolayer and 3D cell culture models. EVs were isolated from plasma samples obtained from patients with OvCa, benign disease, and healthy controls. Targeted miRNA sequencing was conducted on selected groups of miRNAs. Panel (A) shows the miRNA expression across the patient groups, while panel (B) displays the miRNA expression in the groups subclassified by the stage of OvCa. Data are presented as mean ± SE. ∗ (P ≤ 0.05), ∗∗ (P ≤ 0.01), ∗∗∗ (P ≤ 0.001), ∗∗∗∗ (P ≤ 0.0001).

**Fig. 7:**
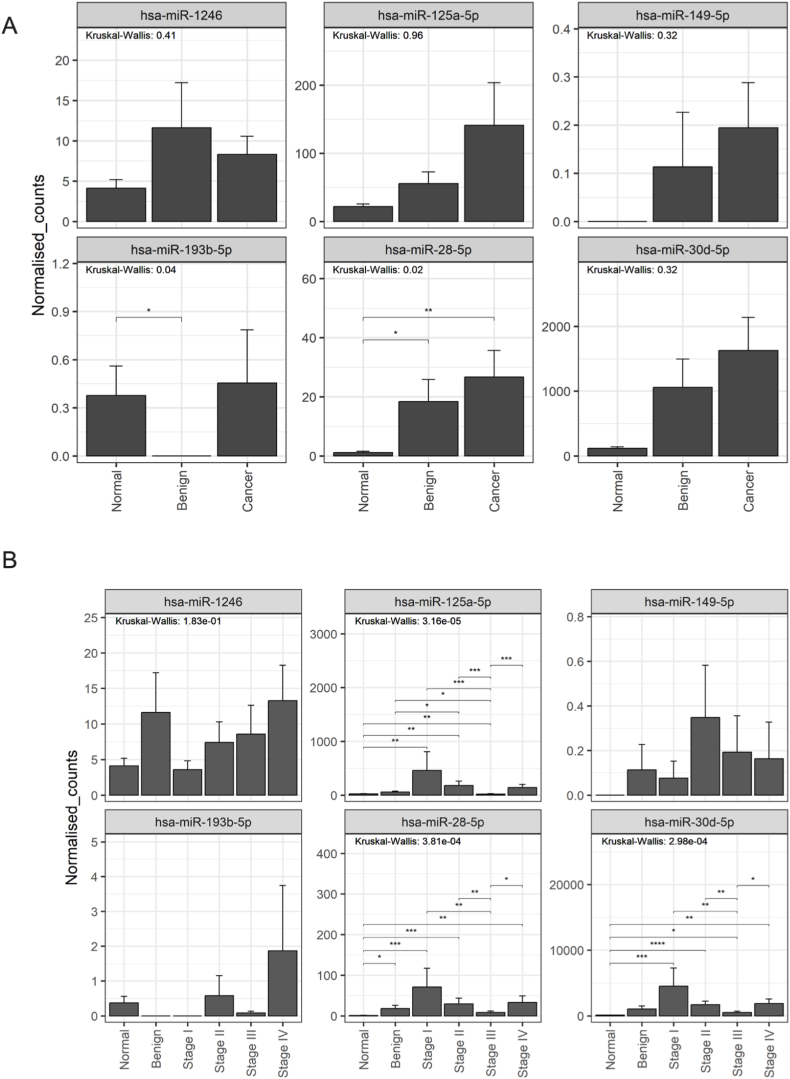
Analysis of miRNAs uniquely identified in extracellular vesicles (EVs) derived from ovarian cancer 3D cell culture models. EVs were isolated from plasma samples obtained from patients with OvCa, benign disease, and healthy controls. Targeted miRNA sequencing was conducted on selected groups of miRNAs. Panel (A) shows the miRNA expression across the patient groups, while panel (B) displays the miRNA expression in the groups subclassified by the stage of OvCa. Data are presented as mean ± SE. ∗ (P ≤ 0.05), ∗∗ (P ≤ 0.01), ∗∗∗ (P ≤ 0.001), ∗∗∗∗ (P ≤ 0.0001).

In Group A (unique to 2D-EVs), hsa-miR-1260b (p ≤ 0.05) and hsa-miR-4508 (p ≤ 0.01) showed significant differences between normal, benign, and cancerous cohorts (Fig. 5A). Further subclassification by cancer stage revealed significant differences in the expression of hsa-let-7a-5p, hsa-let-7g-5p, hsa-let-7i-5p, hsa-miR-1260b, hsa-miR-155-5p, hsa-miR-181a-5p, hsa-miR-182-5p, hsa-miR-186-5p, hsa-miR-30c-5p, hsa-miR-374b-5p, hsa-miR-425-5p, and hsa-miR-4508, compared to normal and benign groups (Fig. 5B). However, no significant differences were found for hsa-miR-3195, hsa-miR-3196, hsa-miR-4454, hsa-miR-4488, and hsa-miR-619-5p. In Group B (shared miRNAs), only hsa-miR-10b-5p exhibited significant differences in expression (p ≤ 0.001) between normal, benign, and cancerous conditions (Fig. 6A). Subclassification into cancer stages revealed significant differences in hsa-let-7b-5p, hsa-let-7f-5p, hsa-miR-10a-5p, hsa-miR-10b-5p, hsa-miR-21-5p, hsa-miR-30a-5p, and hsa-miR-99b-5p (Fig. 6B), while no significant differences were observed for hsa-miR-100-5p, hsa-miR-205-5p, hsa-miR-31-5p, and hsa-miR-320d. In Group C (unique to 3D-EVs), the expression of hsa-miR-193b-5p and hsa-miR-28-5p was significantly different (p < 0.05) between normal, benign, and cancerous conditions (Fig. 7A). A similar subclassification of cancer stages revealed significant differences in hsa-miR-125a-5p, hsa-miR-28-5p, and hsa-miR-30d-5p across the cohort (Fig. 7B). However, no significant differences were found for hsa-miR-1246, hsa-miR-149-5p, and hsa-miR-193b-5p.

**Fig. 8:**
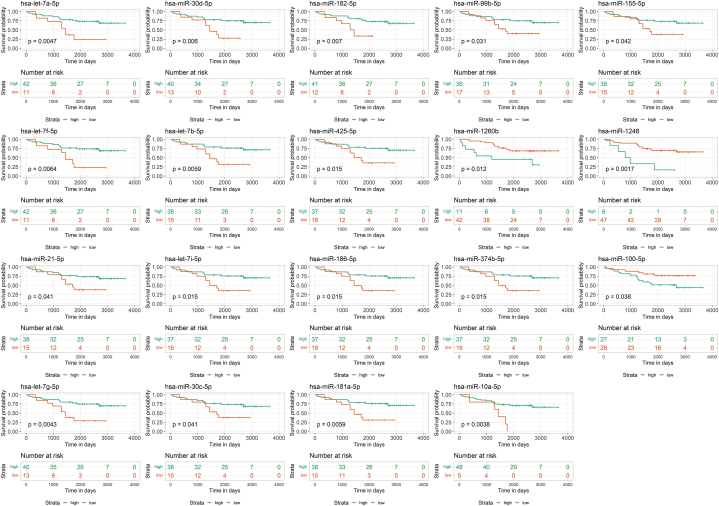
Survival analysis, demonstrating the correlation between the expression of miRNAs within extracellular vesicles and overall survival in ovarian cancer patients. In the plot, red lines represent miRNAs with low expression levels, while green lines represent miRNAs with high expression levels. The x-axis represents the survival time of OvCa patients, while the y-axis indicates the corresponding survival probability.

We also assessed the relationship between overall patient survival and the dysregulated expression of EV-associated miRNAs using Kaplan–Meier analysis, identifying 19 miRNAs significantly associated with survival (p < 0.05) (Fig. 8). Among the miRNAs unique to 2D-EVs, hsa-let-7a-5p, hsa-miR-30c-5p, hsa-let-7i-5p, hsa-miR-182-5p, hsa-let-7g-5p, hsa-miR-181a-5p, hsa-miR-1260b, hsa-miR-186-5p, hsa-miR-425-5p, hsa-miR-155-5p, and hsa-miR-374b-5p were significantly associated with survival. For shared miRNAs, hsa-miR-21-5p, hsa-let-7f-5p, hsa-let-7b-5p, hsa-miR-10a-5p, hsa-miR-100-5p, and hsa-miR-99b-5p were significantly associated with survival. Among miRNAs unique to 3D-EVs, hsa-miR-30d-5p and hsa-miR-1246 were also significantly associated with overall survival.

These findings provide valuable insights into the molecular changes occurring in OvCa cells cultured in 3D and 2D models and pave the way for the development of novel diagnostic and therapeutic strategies targeting EV-associated miRNAs.

## 4 Discussion

Over the past few decades, cancer research has primarily relied on 2D cell cultures to explore the complexity of the tumor microenvironment (TME). While 2D models have been instrumental in uncovering mechanisms of tumor development due to their cost-effectiveness and ease of use, they lack the ability to replicate the full complexity of the TME, particularly the stromal components. This has led to the development of 3D cell culture models that more accurately mimic tissue architecture and the TME, offering a valuable tool for both in vitro and in vivo studies [26].

In this study, we developed a 3D ovarian cancer (OvCa) cell culture model using GelMA hydrogels, a bio-engineered material known for its reproducibility [37]. We selected SKOV-3 and OVCAR-3 cell lines based on their invasive properties [38]. Comprehensive phenotypic characterization, including phase contrast imaging, PicoGreen analysis, and dual fluorescence staining, confirmed the morphology, proliferation, and viability of these cells within the hydrogels. PicoGreen analysis showed significant increases in DNA concentration before serum starvation, indicating that the ECM-based hydrogels support cell proliferation. This was corroborated by histological characterization, where H&E staining revealed multinucleated structures typical of tumors, and immunofluorescence staining confirmed the epithelial-to-mesenchymal transition (EMT), a key hallmark of ovarian cancer [39,40].

EVs isolated from these 3D cultures were characterized following ISEV's MISEV2024 guidelines [28]. SKOV-3 spheroids produced a higher concentration of EVs compared to OVCAR-3, consistent with the invasive nature of SKOV-3 [38,41]. Regardless of the cell line, EVs exhibited heterogeneity in tetraspanin protein expression, reflecting the complex nature of EV populations.

Our study focused on the dynamic changes in EV-associated miRNA signatures derived from 2D and 3D cell cultures and their physiological relevance in ovarian cancer progression. We identified significant differences in miRNA expression between normal, benign, and cancerous conditions, with further subclassification revealing stage-specific alterations. Notably, miRNAs uniquely expressed in 3D cultures, such as hsa-miR-125a-5p, hsa-miR-28-5p, and hsa-miR-30d-5p, were significantly dysregulated in cancer patients compared to normal and benign groups. Of these, dysregulation of hsa-miR-125a-5p and hsa-miR-28-5p has been linked to proliferation, migration, and disease progression in various cancers [[42], [43], [44]]. hsa-miR-30d-5p, identified as a broad-spectrum candidate for diagnostic and therapeutic studies, regulates cellular functions through downstream targets like PI3K/AKT and cyclin E2 (CCNE2), and its dysregulation is linked to tumor development [[45], [46], [47], [48], [49], [50]].

Importantly, hsa-miR-30d-5p exhibited elevated expression in early-stage ovarian cancer (stage I), suggesting its potential as an early diagnostic biomarker. It showed the highest expression among the miRNAs uniquely identified in 3D cultures, further supporting its diagnostic value. Moreover, miRNAs identified in 3D cultures, such as hsa-miR-30d-5p and hsa-miR-1246, were significantly associated with overall patient survival. hsa-miR-30d-5p was uniquely correlated with both early detection and survival, highlighting its dual role as a diagnostic and prognostic biomarker. Interestingly, while hsa-miR-30d-5p was associated with prolonged survival, hsa-miR-1246 was linked to poorer outcomes, reinforcing their distinct roles in disease progression.

Our findings emphasize the need for more physiologically relevant models to better understand the complexities of the TME in ovarian cancer research. The GelMA-based OvCa 3D model used in this study bridges the gap between traditional 2D models and the complex TME, providing new insights into OvCa cell behavior, EV production, and miRNA profiles. Although there are currently no studies that directly explore the role of hsa-miR-30d-5p as a therapeutic candidate, its diagnostic potential cannot be overlooked. This miRNA has shown significant dysregulation in ovarian cancer, particularly in early stages, making it a promising biomarker for early detection. Moreover, emerging technologies such as mature miRNA delivery systems could be leveraged to therapeutically upregulate the expression of hsa-miR-30d-5p. By increasing its expression, it may be possible to suppress key downstream targets involved in tumor progression, including pathways like PI3K/AKT and cyclin E2 (CCNE2), which are linked to increased cell proliferation, migration, and invasion. This approach could open new avenues for developing targeted therapies aimed at slowing or halting tumor development [51].

In conclusion, our study highlights the importance of 3D cell culture models in advancing our understanding of ovarian cancer progression and identifying novel diagnostic and therapeutic strategies. EV-associated miRNAs, particularly those identified in 3D cultures, hold significant potential for predicting survival outcomes in patients with ovarian cancer.

## CRediT authorship contribution statement

**Nihar Godbole:** Writing – review & editing, Writing – original draft, Methodology, Investigation, Formal analysis, Data curation, Conceptualization. **Andrew Lai:** Writing – review & editing, Methodology, Investigation, Formal analysis. **Flavio Carrion:** Writing – review & editing, Investigation, Formal analysis. **Katherin Scholz-Romero:** Supervision, Methodology, Investigation. **Akhilandeshwari Ravichandran:** Validation, Supervision, Methodology, Investigation. **Priyakshi Kalita-de Croft:** Supervision, Methodology, Investigation, Formal analysis. **Amy E. McCart Reed:** Supervision, Resources, Investigation. **Vaibhavi Joshi:** Resources, Investigation. **Sunil R. Lakhani:** Supervision, Resources, Methodology. **Mostafa Kamal Masud:** Resources, Investigation, Formal analysis. **Yusuke Yamauchi:** Writing – review & editing, Supervision, Resources, Data curation. **Lewis Perrin:** Writing – review & editing, Supervision, Resources, Data curation. **John Hooper:** Writing – review & editing, Supervision, Resources. **Laura Bray:** Writing – review & editing, Supervision, Methodology, Investigation, Data curation, Conceptualization. **Dominic Guanzon:** Writing – review & editing, Validation, Supervision, Software, Methodology, Investigation, Formal analysis, Data curation. **Carlos Salomon:** Writing – review & editing, Writing – original draft, Supervision, Resources, Project administration, Funding acquisition, Formal analysis, Data curation, Conceptualization.

## Data availability

All data are available in the main text or the Supplementary Materials and Methods. Further information related to the materials is available from the corresponding author. The small RNA sequencing data is made publicly available and can be accessed using the following link https://doi.org/10.48610/712cbf3.

## Authors’ disclosures

No disclosures were reported by the authors.

## Declaration of competing interest

The authors declare that they have no known competing financial interests or personal relationships that could have appeared to influence the work reported in this paper.
