# Supplementary material for: Extracellular vesicle miRNAs from three-dimensional ovarian cancer in vitro models and their implication in overall cancer survival
Source: Heliyon. 2025 Jan 23;11(4):e42188. doi: 10.1016/j.heliyon.2025.e42188 (PMC11872480; doi:10.1016/j.heliyon.2025.e42188)
Supplement: Multimedia component 1 [file mmc1.docx]

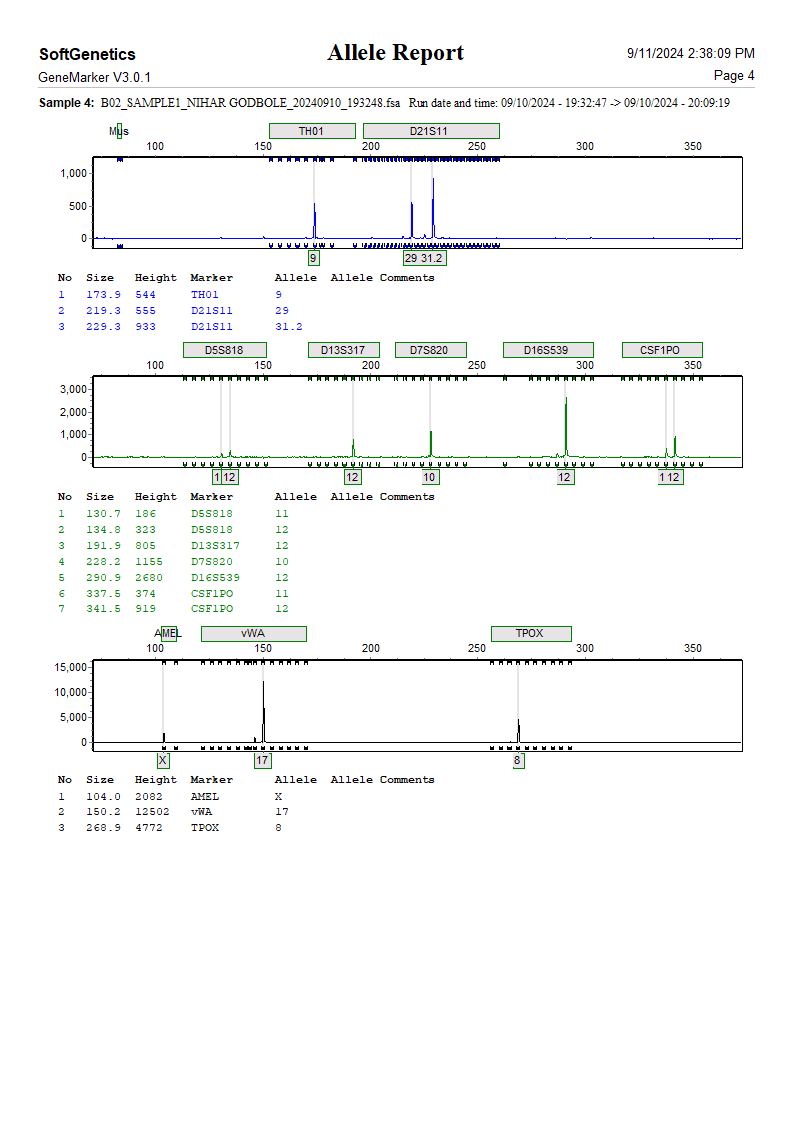
Supplemental Figure 1. OVCAR-3 Cell line authentication.


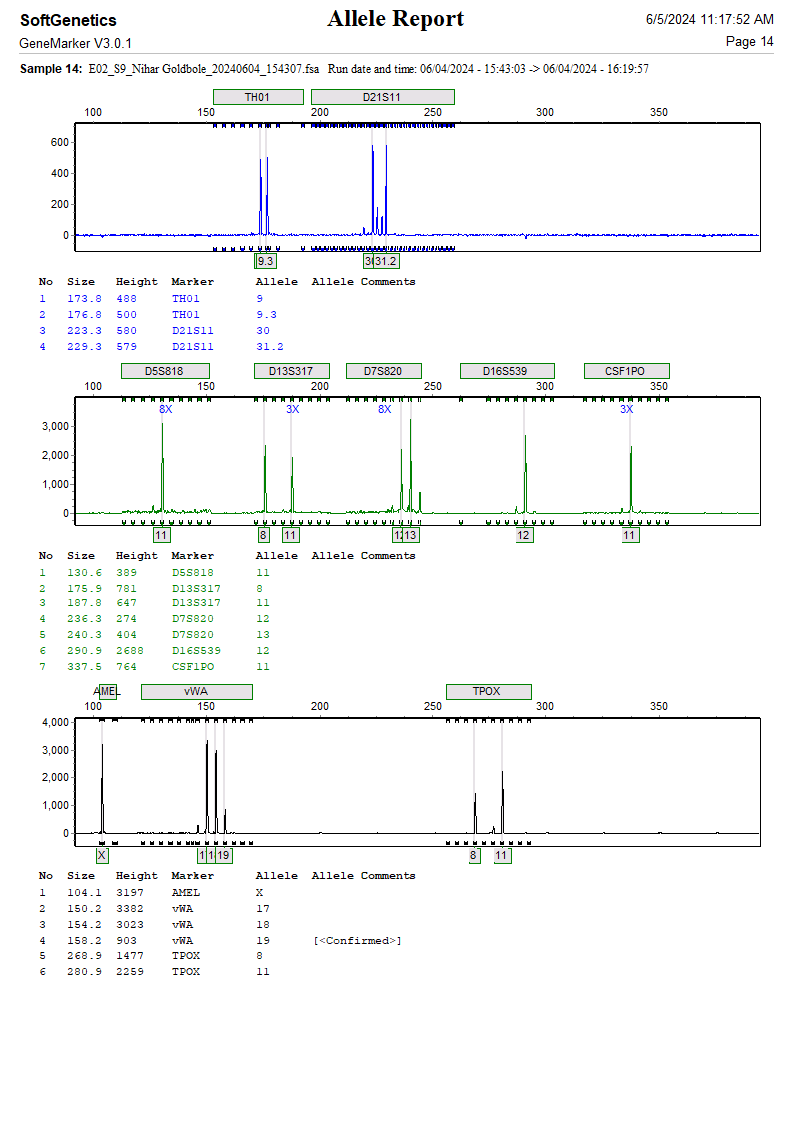


Supplemental Figure 2. SKOV-3 Cell line authentication.


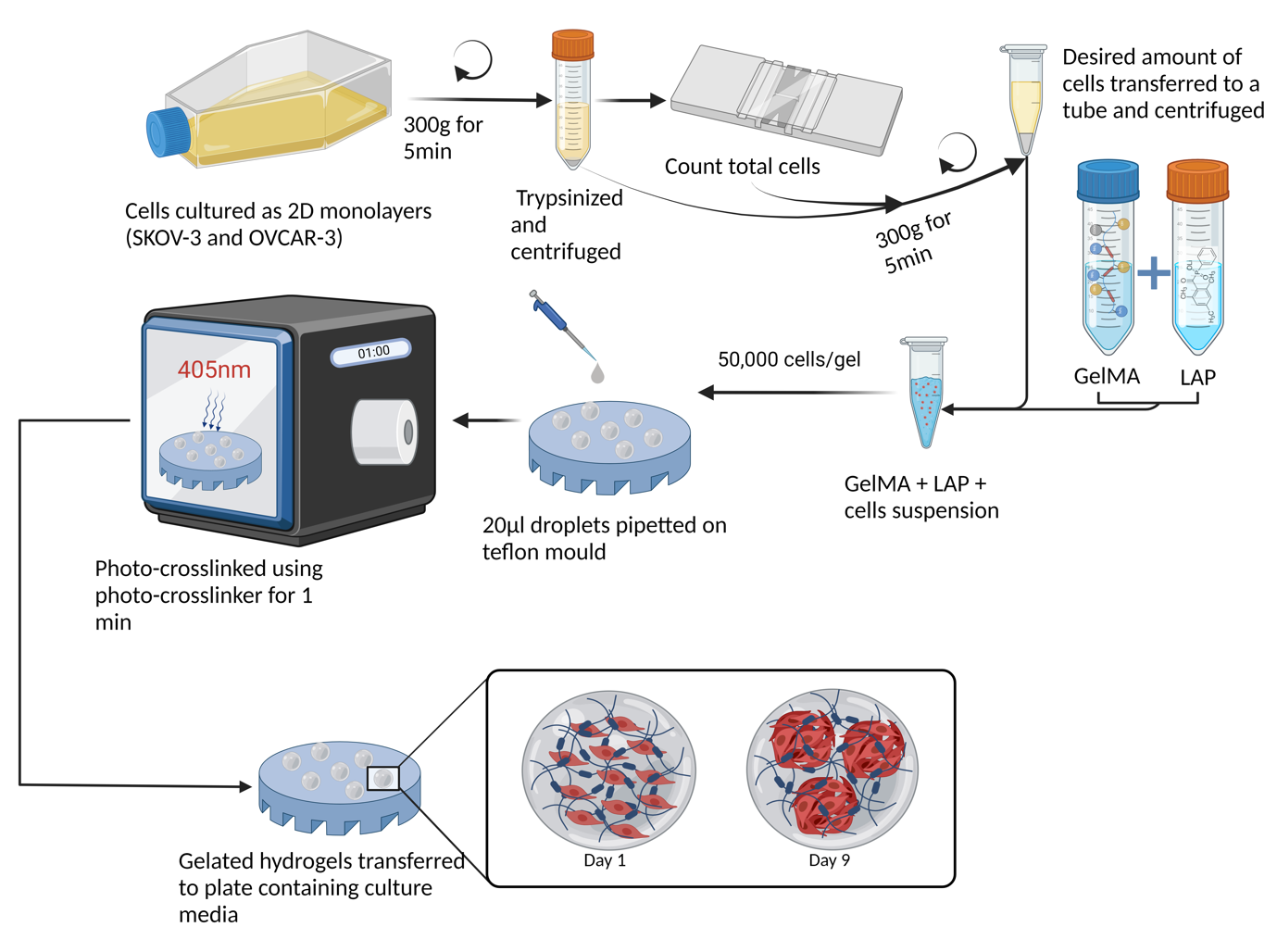


Supplemental Figure 3. Flow diagram describing the Encapsulation using GelMA.


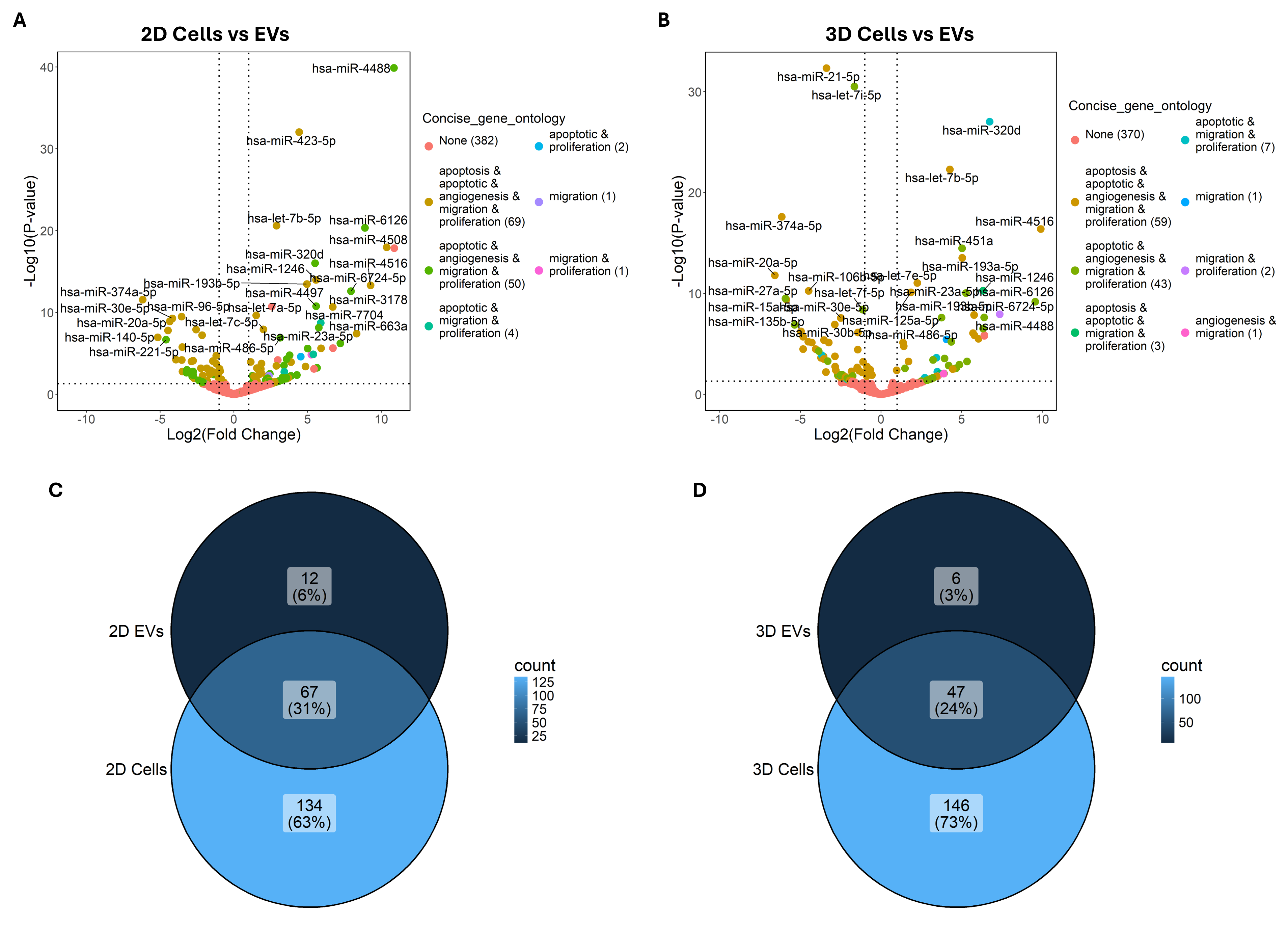


Supplementary Figure 4. Analysis of differentially expressed EV associated miRNAs secreted from ovarian cancer cells cultured in 2D and 3D environments. EVs were isolated from cells cultured in either 2D monolayer or 3D culture models, and their miRNA profiles were determined. The volcano plots illustrate the differential expression of miRNAs from the EVs to their cell counterparts for both 2D and 3D cell cultures of OvCa cells. On the graph, the y-axis represents the log2 transformed fold change, while the x-axis represents negative log10 (p-value). The colours indicate the gene ontology analysis of genes targeted by the miRNAs. (A) Volcano plot illustrating the differential miRNA profiles of the EVs and their cell counterparts for the 2D cell culture model. (B) Volcano plot illustrating the differential miRNA profiles of the EVs and their cell counterparts for the 3D cell culture model. (C) Venn diagram showing the unique and common profiles of miRNAs within the EVs and the OvCa cells in 2D cell culture system. (D) Venn diagram showing the unique and common profiles of miRNAs within the EVs and the OvCa cells in 2D cell culture system.
